# Supplementary material for: Age-specific social mixing of school-aged children in a US setting using proximity detecting sensors and contact surveys
Source: Sci Rep. 2021 Jan 27;11:2319. doi: 10.1038/s41598-021-81673-y (PMC7840989; doi:10.1038/s41598-021-81673-y)

Supplementary Information

Age-specific social mixing of school-aged children in a US setting using proximity detecting sensors and contact surveys

Kyra H. Grantz, Derek A.T. Cummings, Shanta Zimmer, Charles Vukotich Jr., David Galloway, Mary Lou Schweizer, Hasan Guclu, Jennifer Cousins, Carrie Lingle, Gabby M.H. Yearwood, Kan Li, Patti Calderone, Eva Noble, Hongjiang Gao, Jeanette Rainey, Amra Uzicanin, Jonathan M. Read

Supplementary Methods

Proportionate mixing assumptions

Under proportionate mixing assumptions, the probability of any contact being made between individuals in groups *i* and *j* is proportional to the fraction of population in each group:

$$P_{ij} = \begin{cases} \frac{x_i * x_j}{x*(x-1)}, & i \neq j \\ \frac{x_i * (x_j-1)}{x*(x-1)}, & i = j \end{cases}, \quad (1)$$

where x_i is the number of individuals in group *i.*

Assuming a total number of *c* contacts occurs within a population, the number of contacts expected between two groups, $c_{i,j}$, would be:

$$c_{ij} = c * P_{ij} = \begin{cases} c * \frac{x_i * x_j}{x*(x-1)}, & i \neq j \\ c * \frac{x_i * (x_j-1)}{x*(x-1)}, & i = j \end{cases} \quad (2)$$

The total number of contacts expected to be made by an individual in group *i,* c_i, is similarly proportional to the fraction of population in group *i:*

$$c_i = \sum_j c_{ij} = \sum_j \begin{cases} c * \frac{x_i * x_j}{x*(x-1)}, & i \neq j \\ c * \frac{x_i * (x_j-1)}{x*(x-1)}, & i = j \end{cases} \quad (3)$$

$$c_i = c * \frac{x_i}{x*(x-1)} * \sum_j \begin{cases} x_j, & i \neq j \\ (x_j - 1), & i = j \end{cases} \quad (4)$$

$$c_i = c * \frac{x_i}{x*(x-1)} * (x-1) \quad (5)$$

$$c_i = c * \frac{x_i}{x} \quad (6)$$

By substituting equations (2) and (6) for c_{ij} and c_i, respectively, it can be shown that our measure of the age-specific mixing ratio, K_{ij}, reduces to 1 under proportionate mixing:

$$K_{ij} = \begin{cases} \frac{c_{ij}}{c_i * \frac{x_j}{x-1}}, & i \neq j \\ \frac{c_{ij}}{c_i * \frac{x_j - 1}{x-1}}, & i = j \end{cases} \quad (7)$$

$$K_{ij} = \begin{cases} \dfrac{c * \frac{x_i * x_j}{x*(x-1)}}{\left(c* \frac{x_i}{x}\right) * \frac{x_j}{x-1}}, & i \neq j \\ & = 1 \\ \dfrac{c * \frac{x_i*(x_i-1)}{x*(x-1)}}{\left(c* \frac{x_i}{x}\right) * \frac{x_i-1}{x-1}}, & i = j \end{cases} \quad (8)$$

Duration Estimation
Detailed contacts in the middle- and high-school survey could be assigned to one of six categories: less than 10 minutes, 10 to 29 minutes, 30 to 59 minutes, 1 to 2 hours, 2 to 4 hours, or greater than 4 hours. Contacts in the simplified elementary version could be assigned to one of three categories: less than 10 minutes, 10 minutes to 1 hour, or greater than 1 hour.

Each contact was assumed to have a real duration within the reported category (with a maximum length of 24 hours in the highest intervals). An initial duration, in minutes, was randomly assigned within the reported interval from an exponential distribution. The distribution was re-estimated based on these assigned durations, from which new durations were re-assigned. This iterative process was repeated until the updated distribution converged. This procedure was applied to generate total sensor contact durations, using the assumption that each recorded interaction between sensors represented an independent contact of 0 to 20 seconds. One hundred bootstrap replications were performed to generate a single average duration for each contact.

Because deployment lengths differed in each school, we linearly adjusted sensor contact durations to be consistent with a 6-hour deployment, roughly the average length of a school day across the eight participating schools. Because we expected the number of unique contacts to reach saturation early in a given deployment (that is, students do not acquire new contacts at a constant rate, and are instead likely to have made at least one interaction with the majority of their total contacts within the first few hours of a deployment), we made no adjustment for deployment length when calculating unique sensor contacts.

Transmission Model
We used a standard fixed time step Susceptible-Exposed-Infectious-Recovered (SEIR) compartmental model to simulate the spread of respiratory virus through a closed school population.

$$S_{i,t+1} = -S_{i,t} \sum_j \beta K_{ij} \frac{I_{j,t}}{n_j}$$

$$E_{i,t+1} = S_{i,t} \sum_j \beta K_{ij} \frac{I_{j,t}}{n_j} - \gamma E_{i,t}$$

$$I_{i,t+1} = \gamma E_{i,t} - \sigma I_{i,t}$$

$$R_{i,t+1} = \sigma I_{i,t}$$

where $S_{i,t}$ is the number of susceptible individuals in grade *i* at time *t*, $E_{i,t}$ is the number of exposed, but not yet infectious, individuals, $I_{i,t}$ is the number of infectious individuals, and $R_{i,t}$ is the number of recovered individuals. The risk of infection in grade *i* by grade *j* is dependent on

K_{ij}, the measure of contact from group *i* with group *j*, the number of infected individuals in grade *j*, and an effective transmission rate β. $1/\gamma$ is the latent period, and $1/\sigma$ is the period of infectivity. In the short time scale of these models, we assumed no net changes to school population size (including no births, deaths, or changes to enrollment), and no movement between age classes.

We generated the next-generation matrix (1) to define the basic reproductive number, R_0, as a function of the parameters described above. For a given mixing matrix, K, we estimated the effective transmission rate, β, to give an R_0 of 2.0 (2). We note that, in practice, the final outbreak sizes were often smaller than expected (consistent with an R_0 closer to 1.5). The estimation procedure provided an approximation of mean transmission dynamics in which initial infection in any class was expected to make the same contribution to transmission risk. However, as has been reported elsewhere (3), the expected number of secondary cases in an outbreak with identical transmission parameters will decrease as network structure is increased. Following early expansion in certain groups, the epidemic process has difficulty spreading to other groups with fewer contacts and high within-group assortativity, and so fewer infections occur than would be expected in a perfectly connected population. Social mixing results in preferential infection in certain age classes that dynamically changes transmission throughout the outbreak. This phenomenon is particularly evident in the overall lower attack rates in simulations based on sensor-recorded contact matrices with high thresholds of cumulative contact.

References

1. P. Van Den Driessche, J. Watmough, Reproduction numbers and sub-threshold endemic equilibria for compartmental models of disease transmission. *Math. Biosci.* **180**, 29–48 (2002).
2. W. Yang, M. Lipsitch, J. Shaman, Inference of seasonal and pandemic influenza transmission dynamics. *Proc. Natl. Acad. Sci. U. S. A.* **112**, 2723–2728 (2015).
3. L. Danon, T. A. House, J. M. Read, M. J. Keeling, Social encounter networks: Collective properties and disease transmission. *J. R. Soc. Interface* **9**, 2826–2833 (2012).

Supplementary Tables and Figures

Supplementary Table 1. Study population and average number of contacts recorded by self-report contact diaries and proximity detecting mote sensors in a US school setting, by participating school. 6

Supplementary Figure 1. Factors associated with the number and duration of sensor-recorded contact events in a US school setting. All models include a random intercept for deployment day. The number of unique contact events for each participant is defined as the number of other participants with whom the participant had at least 1, 10, or 100 recorded sensor interactions. 8

Supplementary Figure 2. Age-specific mixing matrix of survey-reported in-school contacts in a US school setting as a ratio of observed contacts to expected under proportionate mixing assumptions within each participating school. Blue colors indicate more contacts than expected under proportionate mixing assumptions, and red colors indicate less mixing than expected. Bolded ratio values deviate significantly from the null expectation, α=0.05, and *q* equals the degree of assortative mixing. ELEM, elementary; MS, middle school; HS, high school 9

Supplementary Figure 3. Age-specific mixing matrix of sensor-recorded unique contacts in a US school setting as a ratio of observed contacts to expected under proportionate mixing assumptions within each participating school. Blue colors indicate more contacts than expected under proportionate mixing assumptions, and red colors indicate less mixing than expected. Bolded ratio values deviate significantly from the null expectation, α=0.05, and *q* equals the degree of assortative mixing. ELEM, elementary; MS, middle school; HS, high school 10

Supplementary Figure 4. Age-specific mixing matrix of survey-recorded and sensor-recorded contact durations in a US school setting as a ratio of observed contacts to expected under proportionate mixing assumptions when there is any contact between participants (A, C) and as average per-capita rate of contact (B, D). Blue colors indicate more contacts than expected under proportionate mixing assumptions, and red colors indicate less mixing than expected. Bolded ratio values deviate significantly from the null expectation, α=0.05, and *q* equals the degree of assortative mixing. 11

Supplementary Figure 5. Age-specific mixing matrix of survey-recorded in-school contacts in a US school setting as a ratio of observed contacts to expected under proportionate mixing assumptions using in-school contacts lasting longer than 10 minutes (A), or in-school contacts recorded on a day with a corresponding proximity sensor deployment (B). Blue colors indicate more contacts than expected under proportionate mixing assumptions, and red colors indicate less mixing than expected. Bolded ratio values deviate significantly from the null expectation, α=0.05, and *q* equals the degree of assortative mixing. 12

Supplementary Figure 6. Grade-specific predicted final attack rates of a respiratory virus outbreak in a US school setting, based on stochastic simulation using mixing matrices of in-school survey and unique sensor-recorded contact rates, unadjusted by proportionate mixing expectations, within each school (ELEM, elementary; MS, middle school; HS, high school). 13

Supplementary Figure 7. Grade-specific final predicted attack rates of a respiratory virus in a US school setting, based on stochastic simulation using mixing matrices of in-school survey contacts and unique sensor-recorded contacts at various contact thresholds, adjusted by proportionate mixing expectations, within each school (ELEM, elementary; MS, middle school; HS, high school). (A) R_0 = 1.5; (B) R_0 = 3. 14

Supplementary Table 1. Study population and average number of contacts recorded by self-report contact diaries and proximity detecting mote sensors in a US school setting, by participating school.

Grade	K	1	2	3	4	5	6	7	8	9	10	11	12	Total
Diaries														
No. participants (no. responses)	50 (54)	46 (54)	75 (110)	91 (175)	100 (143)	97 (175)	81 (139)	145 (218)	109 (177)	137 (200)	171 (299)	121 (213)	108 (198)	1331 (2155)
ELEM 1			19 (25)	19 (37)	18 (18)	21 (41)								77 (121)
ELEM 2	15 (15)	14 (14)	22 (42)	33 (64)	31 (59)									115 (194)
ELEM/ MS 1	17 (17)	20 (20)	14 (14)	19 (36)	32 (32)	20 (28)	17 (23)	19 (30)	21 (38)					179 (238)
ELEM/ MS 2	18 (22)	12 (20)	20 (29)	20 (38)	19 (34)	20 (34)	25 (42)	21 (42)	7 (11)					162 (272)
MS 1						36 (72)	38 (73)							74 (145)
MS 2							1 (1)	105 (146)	81 (128)					187 (275)
HS 1										78 (93)	72 (125)	14 (23)	1 (2)	165 (243)
HS 2										59 (107)	99 (174)	107 (190)	107 (196)	372 (667)
No. total contacts, mean (sd)	5.78 (3.39)	11.11 (10.64)	20.5 (25.37)	17.62 (17.3)	20.79 (18.62)	19.82 (34.55)	24.17 (18.14)	34.84 (60.73)	31.34 (48.79)	41.19 (70.41)	28.59 (60.81)	39.89 (106.11)	47.06 (215.45)	29.60 (84.83)
No. in-school contacts, mean (sd)	2.83 (2.27)	1.94 (1.77)	5.06 (5.29)	5.55 (6.17)	8.92 (7.5)	4.7 (6.96)	9.45 (9.19)	11.86 (9.02)	11.98 (9.09)	13.06 (8.68)	9.98 (8.55)	10.21 (8.33)	12.18 (10.1)	9.32 (8.69)

Motes														
No. participants	106	114	87	99	119	230	192	142	113	112	138	96	86	1634
ELEM 1	30	35	26	31	36	38								196
ELEM 2	27	31	22	32	31									143
ELEM/ MS 1	29	33	16	32	28	30	17	20	30					235
ELEM/ MS 2	34	31	36	36	41	33	41	37	32					321
MS 1						144	156							300
MS 2								99	66					165
HS 1										73	58	14	1	146
HS 2										56	87	95	90	328
No. cumulative contacts, mean (sd)	3032.1 (1619)	3069.0 (1418)	2923.7 (1784)	3369.7 (2211)	3808.6 (2421)	3378.5 (1280)	3577.8 (1350)	4061.1 (1407.6)	3232.7 (1762)	2398.8 (1614)	2189.1 (1476)	1896.4 (2216)	1530.7 (855.4)	3068.8 (1795)

sd, standard deviation.

Supplementary Figure 1. Factors associated with the number and duration of sensor-recorded contact events in a US school setting. All models include a random intercept for deployment day. The number of unique contact events for each participant is defined as the number of other participants with whom the participant had at least 1, 10, or 100 recorded sensor interactions.

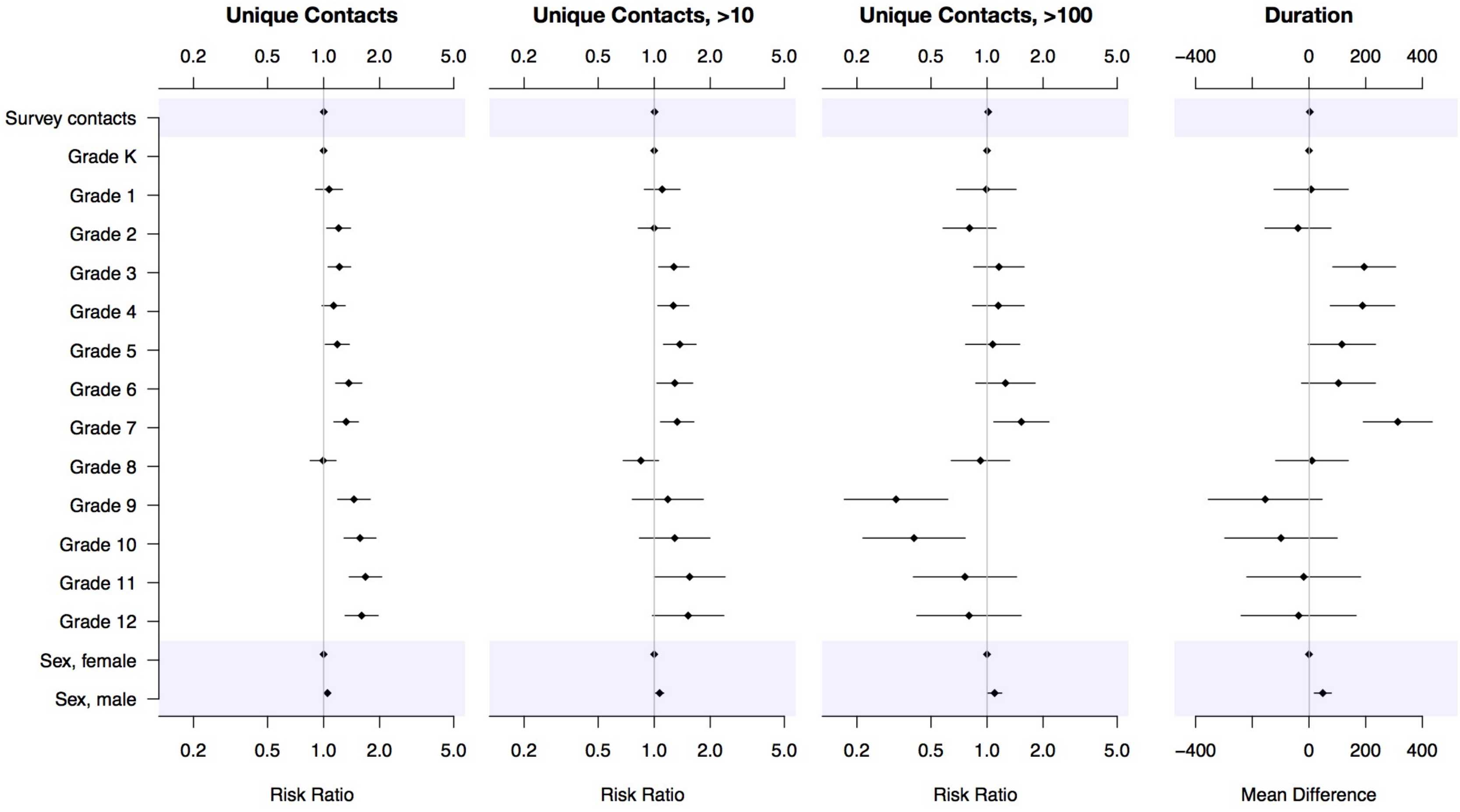

Supplementary Figure 2. Age-specific mixing matrix of survey-reported in-school contacts in a US school setting as a ratio of observed contacts to expected under proportionate mixing assumptions within each participating school. Blue colors indicate more contacts than expected under proportionate mixing assumptions, and red colors indicate less mixing than expected. Bolded ratio values deviate significantly from the null expectation, α=0.05, and *q* equals the degree of assortative mixing. ELEM, elementary; MS, middle school; HS, high school

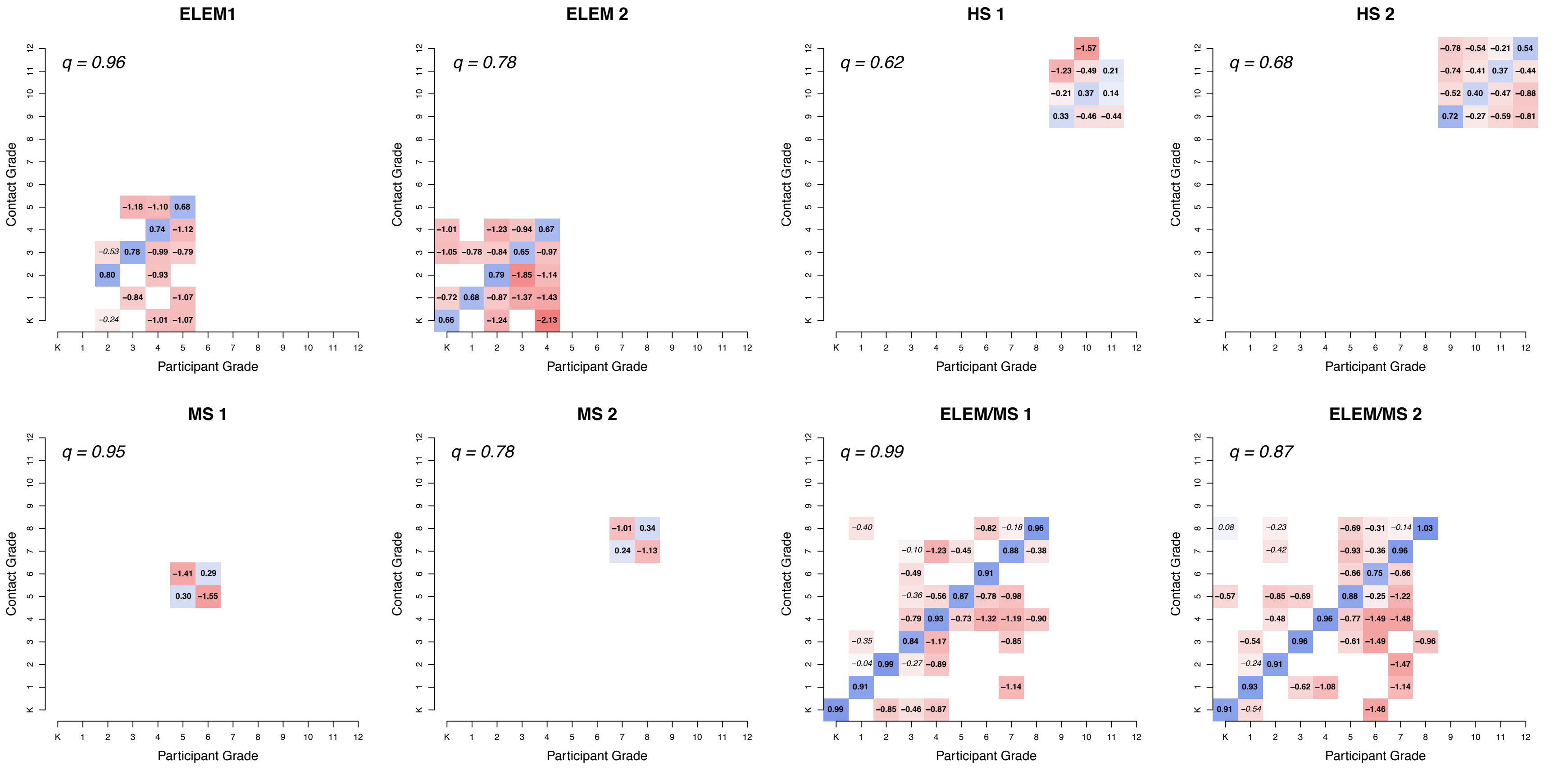

Supplementary Figure 3. Age-specific mixing matrix of sensor-recorded unique contacts in a US school setting as a ratio of observed contacts to expected under proportionate mixing assumptions within each participating school. Blue colors indicate more contacts than expected under proportionate mixing assumptions, and red colors indicate less mixing than expected. Bolded ratio values deviate significantly from the null expectation, α=0.05, and *q* equals the degree of assortative mixing. ELEM, elementary; MS, middle school; HS, high school

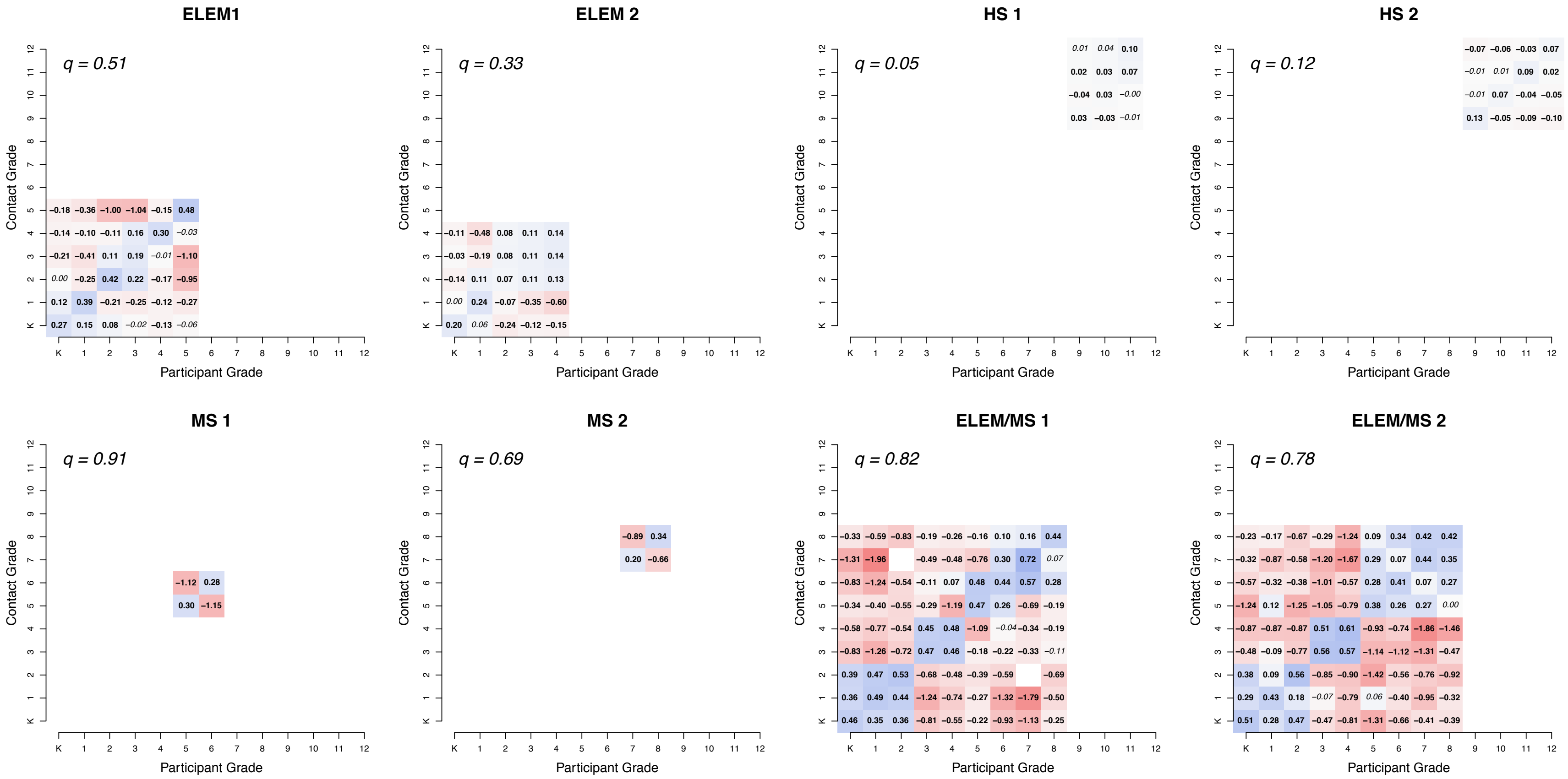

Supplementary Figure 4. Age-specific mixing matrix of survey-recorded and sensor-recorded contact durations in a US school setting as a ratio of observed contacts to expected under proportionate mixing assumptions when there is any contact between participants (A, C) and as average per-capita rate of contact (B, D). Blue colors indicate more contacts than expected under proportionate mixing assumptions, and red colors indicate less mixing than expected. Bolded ratio values deviate significantly from the null expectation, α=0.05, and *q* equals the degree of assortative mixing.

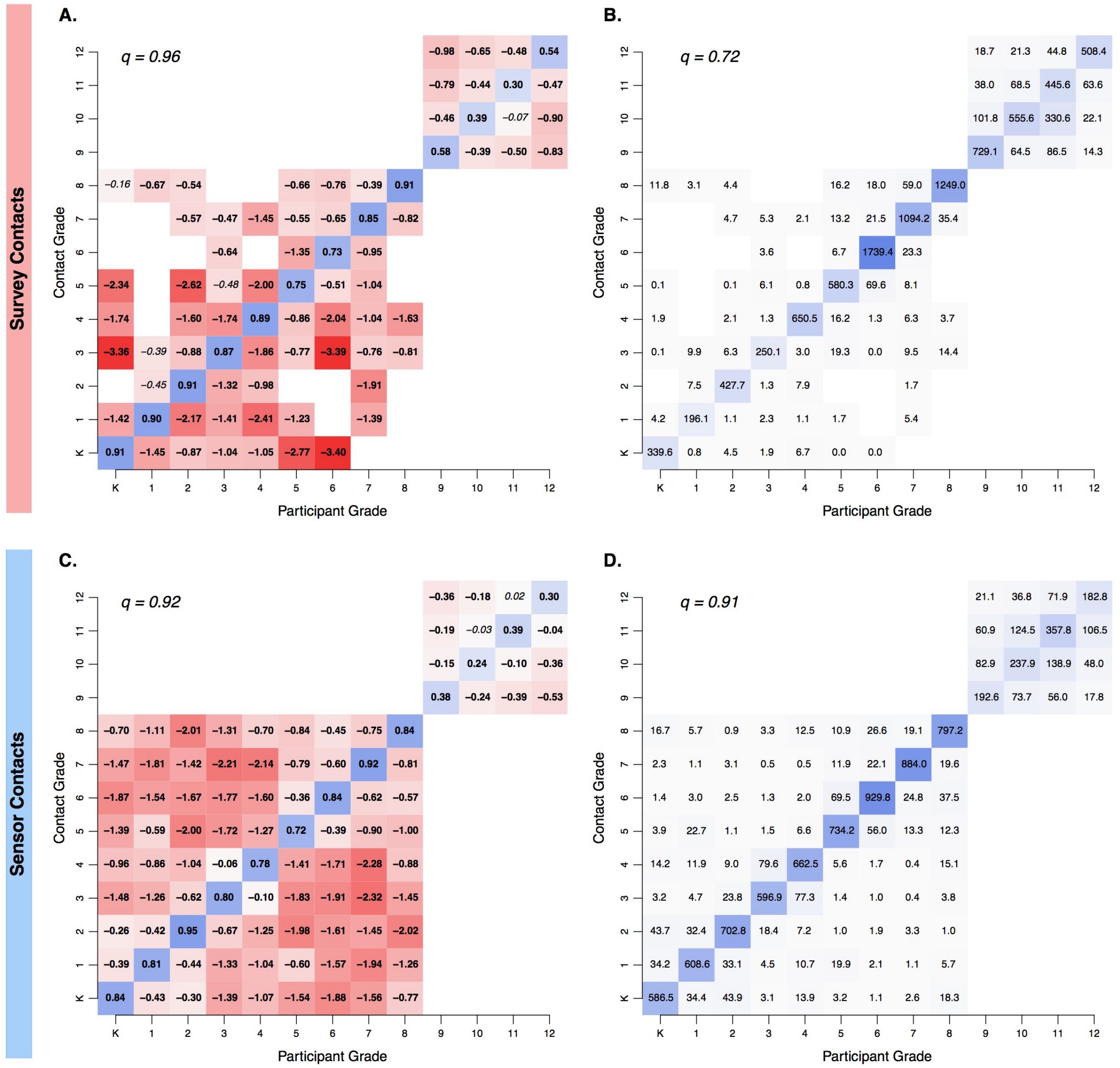

Supplementary Figure 5. Age-specific mixing matrix of survey-recorded in-school contacts in a US school setting as a ratio of observed contacts to expected under proportionate mixing assumptions using in-school contacts lasting longer than 10 minutes (A), or in-school contacts recorded on a day with a corresponding proximity sensor deployment (B). Blue colors indicate more contacts than expected under proportionate mixing assumptions, and red colors indicate less mixing than expected. Bolded ratio values deviate significantly from the null expectation, α=0.05, and *q* equals the degree of assortative mixing.

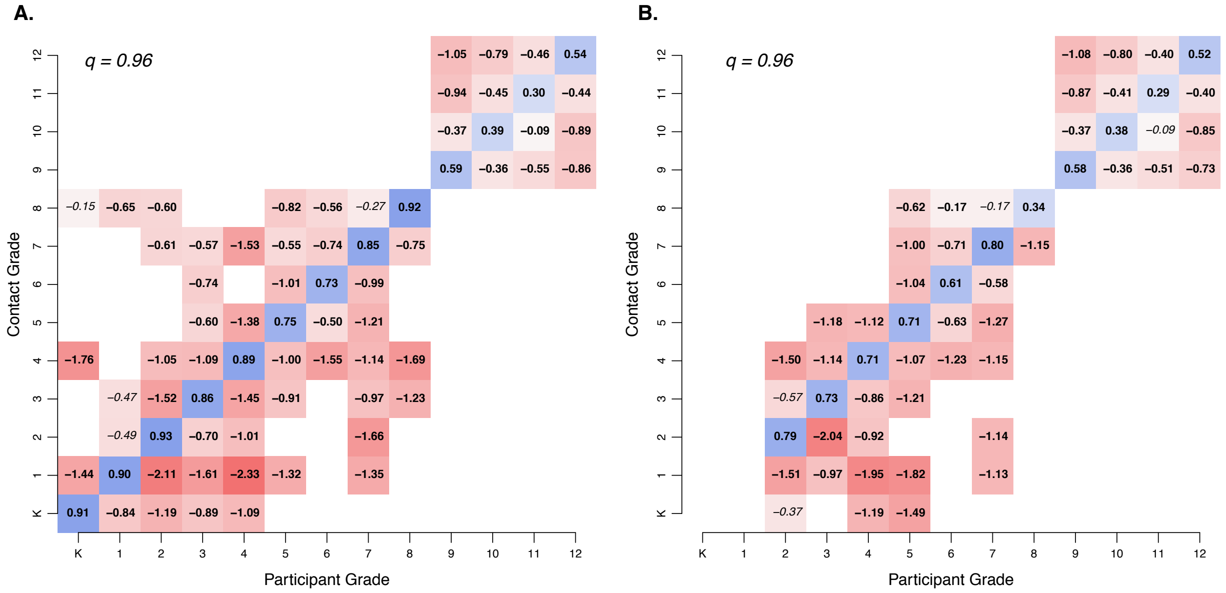

Supplementary Figure 6. Grade-specific predicted final attack rates of a respiratory virus outbreak in a US school setting, based on stochastic simulation using mixing matrices of in-school survey and unique sensor-recorded contact rates, unadjusted by proportionate mixing expectations, within each school (ELEM, elementary; MS, middle school; HS, high school).

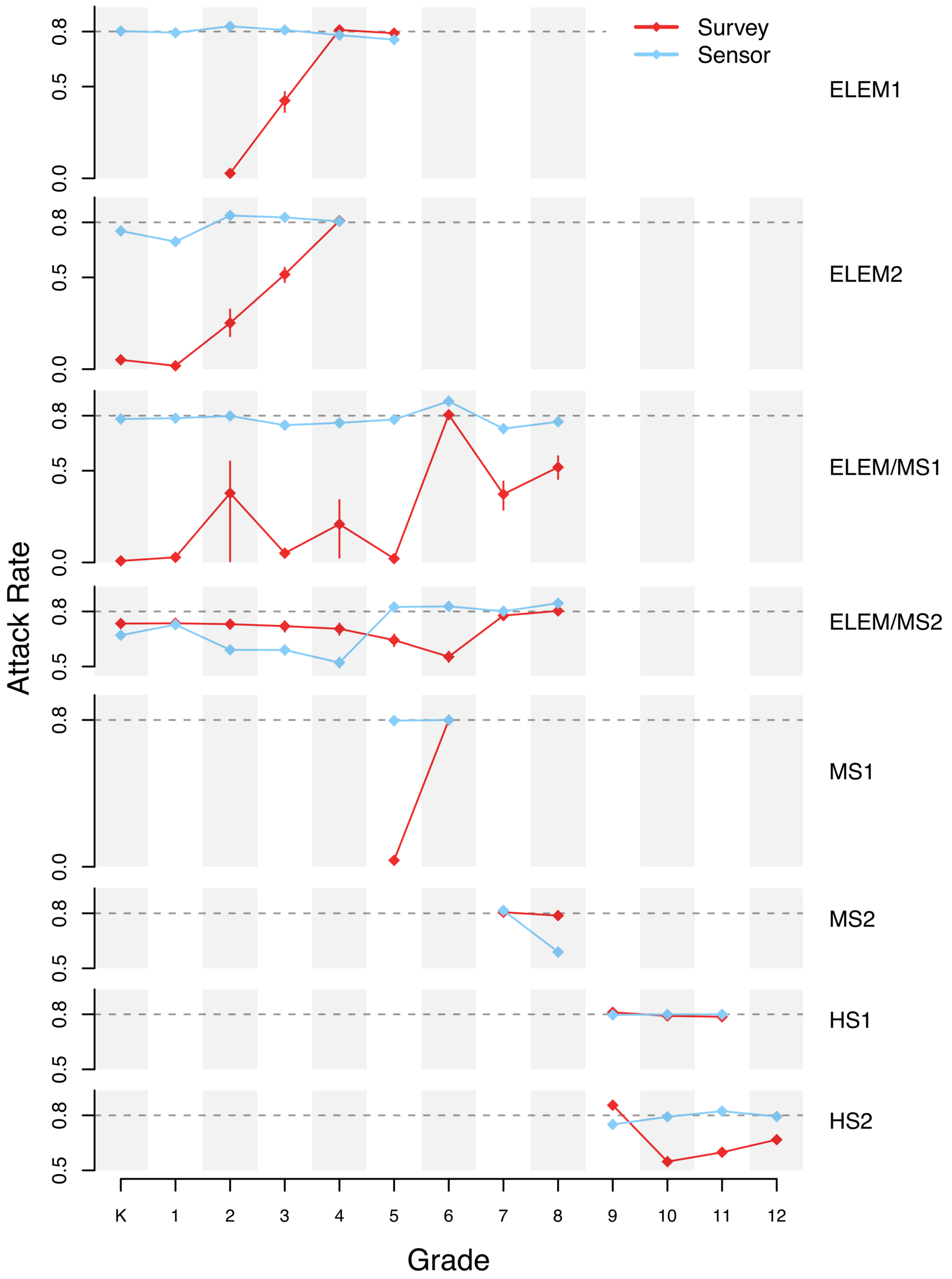

Supplementary Figure 7. Grade-specific final predicted attack rates of a respiratory virus in a US school setting, based on stochastic simulation using mixing matrices of in-school survey contacts and unique sensor-recorded contacts at various contact thresholds, adjusted by proportionate mixing expectations, within each school (ELEM, elementary; MS, middle school; HS, high school). (A) R_0 = 1.5; (B) R_0 = 3.

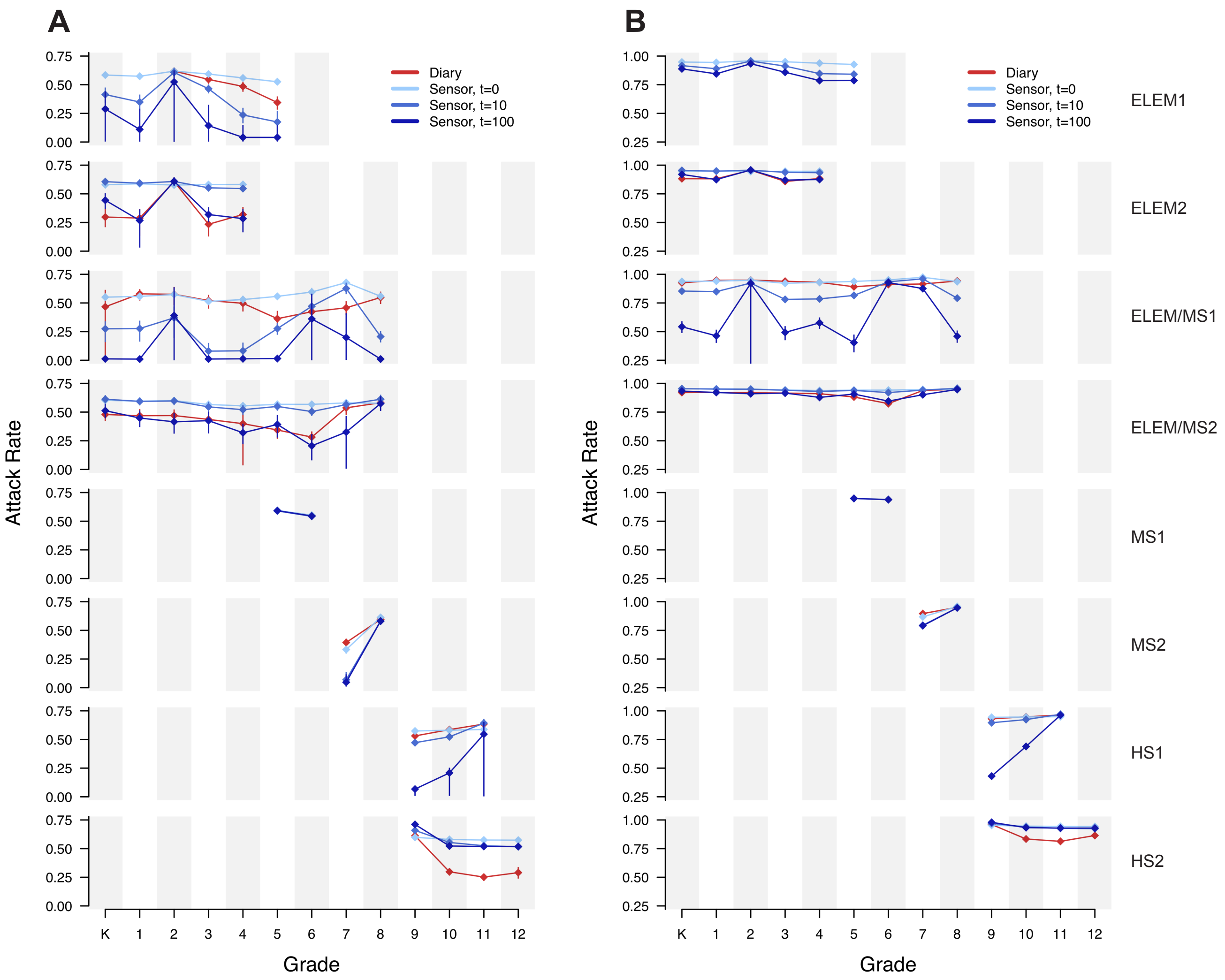

Supplement: Supplementary file 1 — Supplementary Information 1. [file 41598_2021_81673_MOESM1_ESM.pdf]
