# Supplementary material for: Age-specific social mixing of school-aged children in a US setting using proximity detecting sensors and contact surveys
Source: Sci Rep. 2021 Jan 27;11:2319. doi: 10.1038/s41598-021-81673-y (PMC7840989; doi:10.1038/s41598-021-81673-y)
Supplement: Supplementary file 2 — Supplementary Information 2. [file 41598_2021_81673_MOESM2_ESM.pdf]

Appendix 1: Contact survey administered to select participants in elementary schools (grades K to 5), Pittsburgh PA, USA, 2012

Age-specific social mixing of school-aged children in a US setting using proximity detecting sensors and contact surveys

Kyra H. Grantz, Derek A.T. Cummings, Shanta Zimmer, Charles Vukotich Jr., David Galloway, Mary Lou Schweizer, Hasan Guclu, Jennifer Cousins, Carrie Lingle, Gabby M.H. Yearwood, Kan Li, Patti Calderone, Eva Noble, Hongjiang Gao, Jeanette Rainey, Amra Uzicanin, Jonathan M. Read

The SMART study
Social Mixing And Respiratory Transmission in schools

Put your ID sticker in this box

Please write clearly and mark boxes with an 'X'

About You

1 What is your school grade?

K ☐ 1st ☐ 2nd ☐ 3rd ☐ 4th ☐

Put an X in one box, like this ☒

2 What is your school ID code?

☐☐☐☐☐☐☐☐

Write one letter or number in each box, like this 1 2 3 4

3 How old are you?

I am ☐☐ years old

4 Are you a boy or a girl?

a boy ☐ a girl ☐

About your family and home

5 Not counting you, how many people live in your home?

☐☐ people

6 Does any other person sleep in your bedroom?

No ☐ Yes ☐

If yes, how many people sleep in your bedroom, not counting you?

☐☐ people

7 Does anyone in your house go to pre-school or day care?

No ☐ Yes ☐

If yes, how many people go to pre-school or day care?

☐☐ people

8 You go to elementary school. Does anyone else in your house go to elementary school (grades K to 6)?

No ☐ Yes ☐

If yes, how many people?

☐☐ people

9 Does anyone in your house go to middle school (grades 7 or 8)?

No ☐ Yes ☐

If yes, how many people?

☐☐ people

10 Does anyone in your house go to high school (grades 9 to 12)?

No ☐ Yes ☐

If yes, how many people?

☐☐ people

About where you go

11 Did you go anywhere in the last week that is more than one hour's drive away?

No ☐ Yes ☐

About feeling sick and staying away from school

Sometimes when we are sick, we don't go to school. These questions are about the last time you were sick **and** didn't go to school.

12 When you missed school, did you stay at your home?

No ☐ Yes ☐

13 When you missed school, who took care of you?

No-one ☐ Your Mom or Dad ☐

A brother or sister, or someone else you live with ☐ Someone who doesn't live with you ☐

Don't know ☐

About YESTERDAY

The rest of this questionnaire asks about your day YESTERDAY. If you can't remember what you did yesterday, ask your teacher for help.

14 Did you attend school YESTERDAY?

No ☐ Yes ☐

15 If you missed school, why was this?

I was sick or ill ☐

School was closed ☐

Some other reason ☐

16 How did you get to school YESTERDAY?

walked or biked ☐ public bus ☐

by car ☐ some other way ☐

school bus ☐ didn't go to school ☐

Your contact diary

This page asks you about the people you met yesterday

- people you talked with
- people you played with
- people you touched with your hands or face

What to do

step 1 In SECTION A write down the name or nick-name for everyone you met **yesterday**. For example, "mom", "best friend", "mailman". **Write one name in each box.**

step 2 Answer questions 17 to 28 for each of these people in turn.

Do include:

- ✓ children and teachers you spoke to at school
- ✓ children or adults you played with

Do **not** include:

- ✗ people you did not talk to
- ✗ people you only talked with on a telephone
- ✗ pets or toys
- ✗ people you did not meet yesterday

Put your ID sticker in this box

START HERE

Your contacts

Write the name or a description of each person you met yesterday

17 Is this person ... ?
18 Is this person ... ?
19 Does this person go to school?
20 If they go to school ... What grade are they? e.g. K, 7, 12
21 Do they go to your school?
22 Do you live with this person?
23 Where did you meet yesterday? Mark all the places you met them yesterday
24 Did you touch their skin? e.g. holding hands, playing together
25 Did you both hold the same toy or object at all yesterday? e.g. you shared a crayon or hair brush, you swapped food or drink
26 How long did you spend together yesterday?
27 How often do you normally see this person?

#	Name	17 a boy or man	17 a girl or woman	18 a child	18 a grown-up	19 yes	19 no	19 don't know	20 grade	21 yes	21 no	22 yes	22 no	23 at my home	23 at someone else's home	23 at school	23 somewhere else	24 yes	24 no	25 yes	25 no	26 less than 10 min	26 10 min to 1 hour	26 more than 1 hour	27 every day	27 most school days	27 once a week	27 just met them
1		☐	☐	☐	☐	☐	☐	☐		☐	☐	☐	☐	☐	☐	☐	☐	☐	☐	☐	☐	☐	☐	☐	☐	☐	☐	☐
2		☐	☐	☐	☐	☐	☐	☐		☐	☐	☐	☐	☐	☐	☐	☐	☐	☐	☐	☐	☐	☐	☐	☐	☐	☐	☐
3		☐	☐	☐	☐	☐	☐	☐		☐	☐	☐	☐	☐	☐	☐	☐	☐	☐	☐	☐	☐	☐	☐	☐	☐	☐	☐
4		☐	☐	☐	☐	☐	☐	☐		☐	☐	☐	☐	☐	☐	☐	☐	☐	☐	☐	☐	☐	☐	☐	☐	☐	☐	☐
5		☐	☐	☐	☐	☐	☐	☐		☐	☐	☐	☐	☐	☐	☐	☐	☐	☐	☐	☐	☐	☐	☐	☐	☐	☐	☐
6		☐	☐	☐	☐	☐	☐	☐		☐	☐	☐	☐	☐	☐	☐	☐	☐	☐	☐	☐	☐	☐	☐	☐	☐	☐	☐
7		☐	☐	☐	☐	☐	☐	☐		☐	☐	☐	☐	☐	☐	☐	☐	☐	☐	☐	☐	☐	☐	☐	☐	☐	☐	☐
8		☐	☐	☐	☐	☐	☐	☐		☐	☐	☐	☐	☐	☐	☐	☐	☐	☐	☐	☐	☐	☐	☐	☐	☐	☐	☐
9		☐	☐	☐	☐	☐	☐	☐		☐	☐	☐	☐	☐	☐	☐	☐	☐	☐	☐	☐	☐	☐	☐	☐	☐	☐	☐
10		☐	☐	☐	☐	☐	☐	☐		☐	☐	☐	☐	☐	☐	☐	☐	☐	☐	☐	☐	☐	☐	☐	☐	☐	☐	☐
11		☐	☐	☐	☐	☐	☐	☐		☐	☐	☐	☐	☐	☐	☐	☐	☐	☐	☐	☐	☐	☐	☐	☐	☐	☐	☐
12		☐	☐	☐	☐	☐	☐	☐		☐	☐	☐	☐	☐	☐	☐	☐	☐	☐	☐	☐	☐	☐	☐	☐	☐	☐	☐
13		☐	☐	☐	☐	☐	☐	☐		☐	☐	☐	☐	☐	☐	☐	☐	☐	☐	☐	☐	☐	☐	☐	☐	☐	☐	☐
14		☐	☐	☐	☐	☐	☐	☐		☐	☐	☐	☐	☐	☐	☐	☐	☐	☐	☐	☐	☐	☐	☐	☐	☐	☐	☐
15		☐	☐	☐	☐	☐	☐	☐		☐	☐	☐	☐	☐	☐	☐	☐	☐	☐	☐	☐	☐	☐	☐	☐	☐	☐	☐
16		☐	☐	☐	☐	☐	☐	☐		☐	☐	☐	☐	☐	☐	☐	☐	☐	☐	☐	☐	☐	☐	☐	☐	☐	☐	☐
17		☐	☐	☐	☐	☐	☐	☐		☐	☐	☐	☐	☐	☐	☐	☐	☐	☐	☐	☐	☐	☐	☐	☐	☐	☐	☐
18		☐	☐	☐	☐	☐	☐	☐		☐	☐	☐	☐	☐	☐	☐	☐	☐	☐	☐	☐	☐	☐	☐	☐	☐	☐	☐
19		☐	☐	☐	☐	☐	☐	☐		☐	☐	☐	☐	☐	☐	☐	☐	☐	☐	☐	☐	☐	☐	☐	☐	☐	☐	☐
20		☐	☐	☐	☐	☐	☐	☐		☐	☐	☐	☐	☐	☐	☐	☐	☐	☐	☐	☐	☐	☐	☐	☐	☐	☐	☐
21		☐	☐	☐	☐	☐	☐	☐		☐	☐	☐	☐	☐	☐	☐	☐	☐	☐	☐	☐	☐	☐	☐	☐	☐	☐	☐
22		☐	☐	☐	☐	☐	☐	☐		☐	☐	☐	☐	☐	☐	☐	☐	☐	☐	☐	☐	☐	☐	☐	☐	☐	☐	☐
23		☐	☐	☐	☐	☐	☐	☐		☐	☐	☐	☐	☐	☐	☐	☐	☐	☐	☐	☐	☐	☐	☐	☐	☐	☐	☐
24		☐	☐	☐	☐	☐	☐	☐		☐	☐	☐	☐	☐	☐	☐	☐	☐	☐	☐	☐	☐	☐	☐	☐	☐	☐	☐
25		☐	☐	☐	☐	☐	☐	☐		☐	☐	☐	☐	☐	☐	☐	☐	☐	☐	☐	☐	☐	☐	☐	☐	☐	☐	☐
26		☐	☐	☐	☐	☐	☐	☐		☐	☐	☐	☐	☐	☐	☐	☐	☐	☐	☐	☐	☐	☐	☐	☐	☐	☐	☐
27		☐	☐	☐	☐	☐	☐	☐		☐	☐	☐	☐	☐	☐	☐	☐	☐	☐	☐	☐	☐	☐	☐	☐	☐	☐	☐
28		☐	☐	☐	☐	☐	☐	☐		☐	☐	☐	☐	☐	☐	☐	☐	☐	☐	☐	☐	☐	☐	☐	☐	☐	☐	☐
29		☐	☐	☐	☐	☐	☐	☐		☐	☐	☐	☐	☐	☐	☐	☐	☐	☐	☐	☐	☐	☐	☐	☐	☐	☐	☐
30		☐	☐	☐	☐	☐	☐	☐		☐	☐	☐	☐	☐	☐	☐	☐	☐	☐	☐	☐	☐	☐	☐	☐	☐	☐	☐

Put your ID sticker in this box

The SMART study
Social Mixing And Respiratory Transmission in schools

28 Did you meet any more people yesterday that you haven't told us about?

No ☐ Yes ☐

29 If yes, how many more people did you meet yesterday?
Write how many in the boxes

Babies and infants (0-4 years old) ☐☐☐

Children (5-18 years old) ☐☐☐

Grown-ups (19 or older) ☐☐☐

30 Did you meet more or less people yesterday than normal?

Less ☐

About the same ☐

More ☐

31 How easy did you find this questionnaire?

Very easy ☐

Easy ☐

Hard ☐

Very hard ☐

Don't know ☐

32 What was hard about it?
Please write in the box below.

When you have finished and checked your form:

- tear off and keep SECTION A from your contact diary page
- make sure you have put your ID stickers in the boxes on all three pages
- hand all the pages back to your teacher
