# Supplementary material for: Age-specific social mixing of school-aged children in a US setting using proximity detecting sensors and contact surveys
Source: Sci Rep. 2021 Jan 27;11:2319. doi: 10.1038/s41598-021-81673-y (PMC7840989; doi:10.1038/s41598-021-81673-y)
Supplement: Supplementary file 3 — Supplementary Information 3. [file 41598_2021_81673_MOESM3_ESM.pdf]

Appendix 2: Contact survey given to participants in middle and high school (grades 6 to 12) and select participants in elementary schools (grades K to 5), Pittsburgh PA, USA, 2012

Age-specific social mixing of school-aged children in a US setting using proximity detecting sensors and contact surveys

Kyra H. Grantz, Derek A.T. Cummings, Shanta Zimmer, Charles Vukotich Jr., David Galloway, Mary Lou Schweizer, Hasan Guclu, Jennifer Cousins, Carrie Lingle, Gabby M.H. Yearwood, Kan Li, Patti Calderone, Eva Noble, Hongjiang Gao, Jeanette Rainey, Amra Uzicanin, Jonathan M. Read

Put your ID sticker in this box

The SMART study
Social Mixing And Respiratory Transmission in schools

Please write carefully and, where appropriate, mark boxes with an 'X'

About You

1 What is your school grade?

2 What is your school ID code?

Write one letter or number in each box, like this 1 2 3 4

3 How old are you?

I am ___ years old

4 Are you a boy or a girl?

a boy ☐ a girl ☐

Put an X in one box, like this ☒

About your family and home

5 Not counting you, how many people live in your home?

___ people

6 Does any other person sleep in your bedroom?

No ☐ Yes ☐

If yes, how many people sleep in your bedroom, not counting you?

___ people

7 Does anyone in your house go to pre-school or day care?

No ☐ Yes ☐

If yes, how many people go to pre-school or day care?

___ people

8 Does anyone in your house go to elementary school (grades K to 6)?

No ☐ Yes ☐

If yes, how many people?

___ people

9 Does anyone in your house go to middle school (grades 7 or 8)?

Do not count yourself

No ☐ Yes ☐

If yes, how many people?

___ people

10 Does anyone in your house go to high school (grades 9 to 12)?

Do not count yourself

No ☐ Yes ☐

If yes, how many people?

___ people

11 What is the zipcode of your home address?

About where you go

12 Where is the furthest place from your home you went in the past **7** days?

City or town
County
State
Country

13 Where is the furthest place from your home you went in the past **30** days?

City or town
County
State
Country

About feeling sick and staying away from school

Sometimes when we are sick, we don't go to school. These questions are about the last time you were sick **and** didn't go to school.

14 When you missed school, did you stay at your home?

No ☐ Yes ☐

15 When you missed school, who took care of you?

No-one ☐ Your Mom or Dad ☐

A brother or sister, or someone else you live with ☐ Someone who doesn't live with you ☐

Don't know ☐

16 Did you get a flu vaccination this school year?

No ☐ Yes ☐ Don't know ☐

17 Do you believe flu vaccines protect you against the flu?

No ☐ Yes ☐ Don't know ☐

About YESTERDAY

The rest of this questionnaire asks about your day YESTERDAY. If you can't remember what you did yesterday, ask your teacher for help.

18 Did you attend school YESTERDAY?

No ☐ Yes ☐

19 If you missed school, why was this?

I was sick or ill ☐

School was closed ☐

Some other reason ☐

20 How did you get to school YESTERDAY?

walked or biked ☐ public bus ☐

by car ☐ some other way ☐

school bus ☐ didn't go to school ☐

Your contact diary

This page asks you about the people you met yesterday

- people you talked with
- people you played with
- people you touched with your hands or face

Steps to follow

step 1 In SECTION A write down the name or nick-name for everyone you met **yesterday**.
For example, "mom", "Derek", "my best friend", "mailman".
Don't write more than one person's name in each box.

step 2 Answer questions 21 to 32 for each of your contacts.

step 3 Ask your teacher for your two random numbers.
Write these in the spaces at the top of questions 33 and 34
These numbers refer to two of your contacts.
Answer questions 33 and 34 for all of your contacts.

Do include:

- ✓ children and teachers you spoke to at school
- ✓ children or adults you to outside of school
- ✓ people whose skin you touched *(e.g. while playing games)*
- ✓ anyone you briefly spoke with on the way home from school

Do **not** include:

- ✗ people you did not talk to
- ✗ people you only talked with through a telephone or computer
- ✗ pets or toys
- ✗ someone you walked past, but did not speak to or touch their skin
- ✗ people you did not meet yesterday

Put your ID sticker in this box

START HERE

SECTION A

Your contacts

Write the name or a description of each person you met yesterday

21 Is this person ... ?
22 How old is this person? *Enter their age, or your best guess*
23 Does this person go to school?
24 If they go to school ... What grade are they? *e.g. K, 7, 12*
25 Do they go to your school?
26 Do you live with this person?
27 Where did you meet yesterday? *Mark all the places you met them yesterday*
28 Did you touch their skin? *e.g. holding hands, playing together*
29 Did you both hold the same toy or object at all yesterday? *e.g. you shared a crayon or hair brush, you swapped food or drink*
30 How long did you spend together during the whole of yesterday?
31 How often do you normally see this person?
32 Deliberately blank
33 Write your 1st random number here: Did this person meet [contact number] yesterday?
34 Write your 2nd random number here: Did this person meet [contact number] yesterday?

#	Name	21 a boy or man	21 a girl or woman	22 age	23 yes	23 no	23 don't know	24 grade	25 yes	25 no	26 yes	26 no	27 my home	27 a friend's home	27 a relative's home	27 school, in class	27 school, not in class	27 on school bus	27 sports practice or event	27 mall or shopping centre	27 grocery store	27 another type of store	27 restaurant	27 religious service	27 somewhere else	28 yes	28 no	29 yes	29 no	30 less than 10 min	30 10 to 29 min	30 29 to 59 min	30 at least 1 hour, but less than 2	30 at least 2 hours but less than 4	30 4 or more hours	31 every day	31 most school days	31 once a week	31 just met them	33 no	33 probably no	33 probably yes	33 yes	34 no	34 probably no	34 probably yes	34 yes
1																																															
2																																															
3																																															
4																																															
5																																															
6																																															
7																																															
8																																															
9																																															
10																																															
11																																															
12																																															
13																																															
14																																															
15																																															
16																																															
17																																															
18																																															
19																																															
20																																															
21																																															
22																																															
23																																															
24																																															
25																																															
26																																															
27																																															
28																																															
29																																															
30																																															

The SMART study
Social Mixing And Respiratory Transmission in schools

Put your ID sticker in this box

35 Did you meet any more people yesterday that you haven't told us about?

No ☐ Yes ☐

36 If yes, how many more people did you meet yesterday?
Write how many in the boxes

Babies and infants (0-4 years old) ☐☐☐

Children (5-18 years old) ☐☐☐

Grown-ups (19 or older) ☐☐☐

37 Did you meet more or less people yesterday than normal?

Less ☐

About the same ☐

More ☐

38 How easy did you find this questionnaire?

Very easy ☐

Easy ☐

Hard ☐

Very hard ☐

Don't know ☐

39 What was hard about it?
Please write in the box below.

When you have finished and checked your form:

- tear off and keep SECTION A from your contact diary page
- make sure you have put your ID stickers in the boxes on all three pages
- hand all the pages back to your teacher
